# New cryptosporidium genotypes in HIV-infected persons.

**DOI:** 10.3201/eid0503.990318

**Published:** 1999

**Authors:** N J Pieniazek, F J Bornay-Llinares, S B Slemenda, A J da Silva, I N Moura, M J Arrowood, O Ditrich, D G Addiss

**Affiliations:** Centers for Disease Control and Prevention, Atlanta, Georgia 30333, USA. nxp3@cdc.gov

## Abstract

Using DNA sequencing and phylogenetic analysis, we identified four distinct Cryptosporidium genotypes in HIV-infected patients: genotype 1 (human), genotype 2 (bovine) Cryptosporidium parvum, a genotype identical to C. felis, and one identical to a Cryptosporidium sp. isolate from a dog. This is the first identification of human infection with the latter two genotypes.


### New *Cryptosporidium* Genotypes in HIV-Infected Persons

**Norman J. Pieniazek,* Fernando J. Bornay-Llinares,* Susan B. Slemenda,* Alexandre J. da Silva,* Iaci N. S. Moura,* Michael J. Arrowood,* Oleg Ditrich,† and David G. Addiss***
*Centers for Disease Control and Prevention, Atlanta, Georgia, USA; and
†Institute of Parasitology AS CR, České Budějovice, Czech Republic

Using DNA sequencing and phylogenetic analysis, we identified four distinct *Cryptosporidium* genotypes in HIV-infected patients: genotype 1 (human), genotype 2 (bovine) *Cryptosporidium parvum*, a genotype identical to *C. felis*, and one identical to a *Cryptosporidium* sp. isolate from a dog. This is the first identification of human infection with the latter two genotypes.

Protozoan apicomplexan parasites from the genus *Cryptosporidium* infect a wide variety of hosts (1). The parasites are transmitted to humans through contaminated drinking water (2), contact with infected animals, and contact with infected persons (3). In the immunocompetent, cryptosporidiosis manifests itself as self-limited diarrhea, sometimes accompanied by nausea, abdominal cramps, fever, and vomiting. In the immunodeficient, however, cryptosporidiosis may be severe, chronic, and life-threatening (4).

Cross-infection experiments, in which *Cryptosporidium* oocysts were obtained from animals of one species and fed to animals of another species, have investigated the host specificity of this parasite (5). The differences observed in the host range of putative *C. parvum* isolates led to a proposal to establish the *Cryptosporidium* isolate originating from guinea pigs, morphologically indistinguishable from *C. parvum*, as a new species—*C. wrairi*—solely on the basis of experimental infection (6). The possibility of many *Cryptosporidium* species fostered the development of techniques suitable for typing isolates. Commonly used techniques are isoenzyme analysis (7), Western blotting (8,9), random amplified polymorphic DNA analysis (10-12), polymerase chain reaction restriction fragment length polymorphism (PCR-RFLP) analysis (13,14), and PCR followed by DNA sequencing (11,15-19).

Address for correspondence: Norman J. Pieniazek, Division of Parasitic Diseases, Centers for Disease Control and Prevention, 4770 Buford Highway NE, Mail Stop F13, Atlanta, GA, 30341-3724, USA; fax: 770-488-4108; e-mail: nxp3@cdc.gov.

Early studies of the polymorphism of isolates classified as *C. parvum* found significant geographic variation among isolates (20) in the region coding for the small subunit ribosomal RNA (SSU-rRNA), commonly used for taxonomic classification. Recently, it has been shown (21) that one of the sequences used in this analysis (22) was erroneously identified as a *C. parvum* sequence, while in fact it was *C. muris*. More recent work (e.g., Le Blancq et al. [23] and GenBank entry AF040725) has shown that the SSU-rRNA region of the *C. parvum* zoonotic (bovine) genotype does not show heterogeneity and is practically identical to the sequence submitted to GenBank in 1993 (accession number L16996, [24,25]).

Recently, consistent results of typing bovine and human *C. parvum* isolates led to unequivocal recognition of two genotypes of *C. parvum*. These two genotypes were reproducibly differentiated by sequencing the SSU-rRNA coding region (16), sequencing the *Cryptosporidium* thrombospondin-related adhesion protein (TRAP-C2) gene (17), and PCR-RFLP analysis of the *Cryptosporidium* oocyst wall protein (COWP) gene (15). Thus far, the anthroponotic genotype (genotype 1) has been found only in infected humans, while the zoonotic genotype (genotype 2) has been found both in infected humans and in livestock, e.g., cows, lambs, goats, and horses. The published partial SSU-rRNA sequence of the *C. parvum* genotype 1 (16) is identical to the *Cryptosporidium* SSU-rRNA sequence that we observed in PCR-amplified DNA from a patient with AIDS (GenBank accession number L16997). That the data from different laboratories for these two genotypes were reproducible calls into question the recent conclusion of Tzipori and Griffiths (26) that "there are no clearly defined and fully characterized reference 'strains' of *Cryptosporidium*."

Other new *Cryptosporidium* genotypes have been identified in various animals (pigs, mice, cats, and koalas) by sequencing the region coding for SSU-rRNA (16,27,28). The cat genotype is thought to represent *C. felis* (28). When morphologic and other data are available for these new genotypes, they may be recognized as new *Cryptosporidium* species.

We present results of typing *Cryptosporidium* isolates by direct sequencing the variable region of the SSU-rRNA amplified from fecal specimens with *Cryptosporidium* genus-specific diagnostic PCR primers (25). Applying this method to a set of specimens from a 3-year longitudinal study on the risk for enteric parasitosis and chronic diarrhea in immunodeficient patients with a low CD4+ count, we found the first human cases of infection by *C. felis* and by a newly identified zoonotic *Cryptosporidium* species possibly originating from a dog.

### The Study

All analyzed specimens were collected from January 1991 through September 1994 in a study assessing the impact of enteric parasite-associated diarrhea in persons infected with HIV (29). Study participants answered comprehensive questionnaires concerning clinical and epidemiologic information and provided stool specimens monthly. All stool specimens were examined for *C. parvum* by Kinyoun carbol-fuchsin modified acid-fast stain (30) and direct immunofluorescence (31). Stained slides were examined by observing 200 oil-immersion fields. *C. parvum* was associated with 18 (5.1%) of the 354 acute episodes and 36 (12.9%) of the chronic episodes of diarrhea reported by the participants. All specimens from this study were preserved in separate vials of 10% formalin and polyvinyl alcohol (Para-Pak Stool System; Meridian Diagnostics, Inc., Cincinnati, OH) and thus were not suitable for molecular analysis. Some specimens were originally aliquoted and stored without preservation at -80°C. Of this set, we selected 18 available specimens for 10 randomly chosen *Cryptosporidium*-positive patients for this study.

An aliquot of approximately 300 µl of each stool specimen was suspended in 1 ml of 0.01 M phosphate-buffered saline, pH 7.2, containing 0.01 M of EDTA (PBS-EDTA), and the suspension was centrifuged at 14,000 x g, 4°C for 5 minutes. The pellet from this centrifugation was washed two more times under the same conditions. The pellet was resuspended in 300 µl of PBS-EDTA and used for DNA extraction, performed with the FastPrep disrupter and the FastDNA kit (BIO 101, Inc., Vista, CA) (32). Extracted DNA was stored at 4°C until PCR amplification.

*Cryptosporidium* genus-specific primers (CPBDIAGF and CPBDIAGR) were used to amplify the *Cryptosporidium* SSU-rRNA variable region (24,25). The conditions for PCR were 95°C for 15 minutes; 45 cycles of denaturation at 94°C for 30 seconds, annealing at 65°C for 30 seconds, extension at 72°C for 1 minute, 30 seconds; followed by extension at 72°C for 9 minutes; and finished with a hold step at 4°C.

PCR products were analyzed by electrophoresis on 2% SeaKem GTG agarose (Cat. No. 50074, FMC Bioproducts, Rockland, ME), stained with ethidium bromide, and visualized on UV transilluminator. The size of the diagnostic fragment amplified with these primers for *C. parvum* genotype 1 (GenBank accession number L16997) was 438 bp, for *C. parvum* genotype 2 (L16996) 435 bp, for *C. baileyi* (L19068) 428 bp, and for *C. muris* (L19069) 431 bp.

PCR products were purified by using the Wizard PCR Preps kit (Cat. No. A7170, Promega, Madison, WI). Sequencing reactions were done with the Perkin Elmer Big Dye kit (Cat. No. 4303149, PE Biosystems, Foster City, CA) and analyzed on the Perkin Elmer ABI 377 automatic DNA sequencer. Sequences were assembled by using the program SeqMan II (DNASTAR Inc., Madison, WI). Sequences were aligned with the program MACAW (33) and analyzed by programs from the PHYLIP (phylogeny inference program) package (34).

### Findings

After the 18 fecal specimens were retrieved from storage and processed for PCR with the

CPBDIAGF/CPBDIAGR diagnostic primer set, visualization of PCR products on 2% agarose gels found two different sizes of diagnostic bands. The size of the *Cryptosporidium* diagnostic bands for patients 53, 75, 119, 124, 153, 278, and 554 did not differ significantly from the size of the standard band (approximately 435 bp) obtained with cloned SSU-rRNA for the *C. parvum* genotype 2 (Figure 1, lane 1). On the other hand, the *Cryptosporidium* diagnostic bands in samples from patients 84, 184, and 485 were visibly larger, approximately 450 bp (Figure 1, lane 3 shows data for patient 84).

DNA sequence analysis showed four types of sequences (Table 1). The sequence of the diagnostic band from patient 53 was 435 bp long and was identical to the corresponding fragment

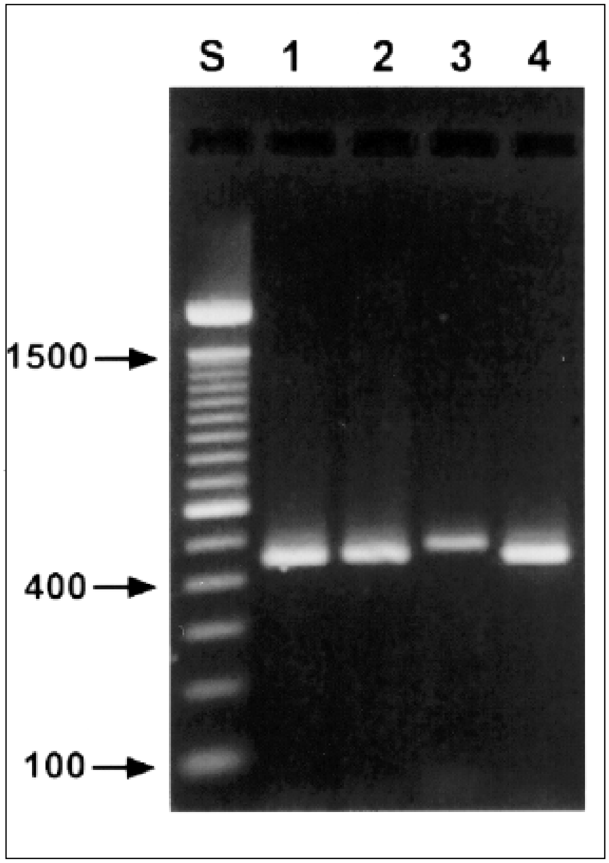

Figure 1. Agarose gel (2%) visualization of diagnostic polymerase chain reaction products of four *Cryptosporidium* genotypes. Lane S, standard 100-bp ladder; lane 1, patient 53, zoonotic genotype 2; lane 2, patient 119, *Cryptosporidium* sp. (zoonotic, canine genotype); lane 3, patient 84, *C. felis* (zoonotic, feline genotype); lane 4, patient 75, anthroponotic genotype 1.

Table 1. *Cryptosporidium* genotypes for 10 selected patients in this study

| Patient no. | Band size[a] (bp) | *C. parvum* genotype |
|---|---|---|
| 53 | 435 | Zoonotic (genotype 2) |
| 75, 124, 153 278, 554 | 438 | Anthroponotic (genotype 1) |
| 119 | 429 | *Cryptosporidium* sp. (canine genotype) |
| 84, 184, 485 | 455 | *C. felis* |

[a]Size of the *Cryptosporidium* diagnostic band obtained with the CPBDIAGF/CPBDIAGR PCR primer pair of the *C. parvum* genotype 2 (GenBank accession number L16996) SSU-rRNA. The diagnostic bands in patients 75, 124, 153, 278, and 554 were 438 bp long and were identical to the corresponding fragment of the *C. parvum* genotype 1 SSU-rRNA (GenBank accession number L16997). The sequence from patient 119 was 429 bp long; the diagnostic bands from patients 84, 184, and 485 were 455 bp long and were identical to each other. Sequence similarity searches of GenBank database and molecular phylogeny analysis of the two latter types of sequences showed that, while they were not identical to any sequences in GenBank, they clustered within other representative SSU-rRNA sequences from the genus *Cryptosporidium* (results not shown). In addition, these sequences were significantly different from recently reported partial SSU-rRNA sequences (16,19,27,28). The 429-bp-long diagnostic fragment in patient 119 was identical to a recently identified canine *C. parvum* isolate SSU-rRNA sequence (GenBank accession number AF112576). The longest sequence (455 bp) from patients 84, 184, and 495 was identical in a 100-bp overlap to an updated sequence (GenBank accession number AF097430) for *C. felis* (also called feline *C. parvum* genotype) (28). Alignment of these sequences is shown in Figure 2. In the hypervariable alignment region (from position 101 to 110), all genotypes display a variable number of thymidines. Genotype 1 has the longest stretch, 11 thymidines, while the *Cryptosporidium* sp. (canine genotype) has only one. The sequence for *C. felis* has five insertions at alignment positions 45, 86, 111, 187, and 218, as well as one deletion of two thymidines at positions 197 and 198. Apart from the difference in the hypervariable region, the *Cryptosporidium* sp. (canine genotype) has a deletion of two bases at positions 49 and 50.

Figure 2. Alignment of the *Cryptosporidium* small subunit ribosomal DNA (SSU-rRNA) diagnostic fragments obtained with the CPBDIAGF/CPBDIAGR polymerase chain reaction primer pair for the four genotypes. Only the first 300 columns of the alignment are shown, as the remaining columns were identical for all genotypes. Gaps are shown with dashes (-), and bases identical to the base in the first row (*C. felis*) are shown with dots (.). Numbers to the right of the alignment show sequence positions for each genotype. The sequences for the SSU-rRNA diagnostic fragment of all four genotypes were submitted to GenBank and were assigned accession numbers AF087574, AF087575, AF087576, and AF087577.

Results of genotyping the 18 specimens are in Table 2. For patients 53, 124, 153, 184, 278, and 485, only a single specimen was available. For other patients (e.g., patient 554), four specimens collected during 12 months were available. The same *Cryptosporidium* genotype persisted throughout a patient's infection.

### Conclusions

Using the sequence of a diagnostic fragment of SSU-rRNA, as well as two well-established genotypes of *C. parvum* (anthroponotic genotype 1 and zoonotic genotype 2), we detected two new *Cryptosporidium* genotypes. The first, in patients 84, 184, and 485, was identical to the

Table 2. Persistence of *Cryptosporidium* genotypes in patients[a]

| Patient no. | No. of available specimens | Month of follow-up | | | | | | | | | | | |
|---|---|---|---|---|---|---|---|---|---|---|---|---|---|
| | | 1 | 2 | 3 | 4 | 5 | 6 | 7 | 8 | 9 | 10 | 11 | 12 |
| 53 | 1 | B | | | | | | | | | | | |
| 75 | 2 | H | - | - | H | | | | | | | | |
| 84 | 4 | F | - | F | - | F | F | | | | | | |
| 119 | 2 | C | - | C | | | | | | | | | |
| 124 | 1 | H | | | | | | | | | | | |
| 153 | 1 | H | | | | | | | | | | | |
| 184 | 1 | F | | | | | | | | | | | |
| 278 | 1 | H | | | | | | | | | | | |
| 485 | 1 | F | | | | | | | | | | | |
| 554 | 4 | H | - | - | - | - | - | - | H | - | H | - | H |

[a]B, *C. parvum* genotype 2 (zoonotic, bovine); C, *Cryptosporidium* sp. (zoonotic, canine); F, *C. felis* (zoonotic, feline); H, *C. parvum* genotype 1 (anthroponotic); -, specimen not available.

feline *Cryptosporidium* genotype (28), also described as *C. felis*. The second, in two specimens from patient 119, represents the newly identified *Cryptosporidium* sp. found in a sequence originating from a dog (GenBank accession number AF112576).

New taxons may be established within the genus *Cryptosporidium* on the basis of the isolate's host range together with molecular data, even though morphologic criteria are apparently lacking. We propose to use *C. felis* Iseki, 1979, instead of feline *C. parvum* genotype; we believe that the anthroponotic genotype 1 of *C. parvum* and the *Cryptosporidium* sp. (canine genotype) described here should be named as distinct species. Although the taxonomy of protists is morphology-based, the taxonomy of bacteria is being reevaluated on the basis of molecular data (35). There is no reason to use different approaches to these two kingdoms, nor is it necessary to postulate that the basis for speciation of *Cryptosporidium* remains ambiguous or that molecular data lend little support to separation of *Cryptosporidium* into distinct, valid species (26). Further, we see no evidence for the unconventional conclusions of Tzipori and Griffiths that "...genetic markers in *C. parvum* can change upon passage to a different host, possibly through a selective mechanism favoring different populations" (26) nor for those of Widmer (21) that "...genetic studies and experimental infections suggest that a selective mechanism triggered by a change in the intestinal environment might be involved in shaping the genetic make-up of *C. parvum* populations."

The finding of new *Cryptosporidium* genotypes in immunodeficient patients might suggest a unique susceptibility to infections by divergent *Cryptosporidium* species circulating in companion animals or livestock. However, recent identification of *C. felis* in a cow (36) may indicate a complex pattern of flow of different *Cryptosporidium* species in the environment. When more data on the distribution of these species become available, public health and preventive measures will need to be reevaluated, and isolates may need to be renamed to reflect their natural history. Finally, it is clear that Kinyoun carbol-fuchsin modified acid-fast stain (30) and direct immunofluorescence (31) could detect all genotypes, but this may not be true for diagnostic reagents that may be affected directly (PCR) or indirectly (Ab) by the genetic composition of the new *Cryptosporidium* isolates reported here. Thus, discovery of these new *Cryptosporidium* genotypes in human cryptosporidiosis should cause existing diagnostic tools to be reevaluated.

### Acknowledgment

The authors thank Jane L. Carter for her expert help with running the DNA sequencer.

Dr. Pieniazek is a microbiologist with the National Center for Infectious Diseases, CDC. His research interests include molecular reference diagnosis of parasitic diseases and molecular systematics.

### References

1. Fayer R, Speer CA, Dubey JP. The general biology of *Cryptosporidium*. In: Fayer R, editor. *Cryptosporidium* and cryptosporidiosis. Boca Raton (FL): CRC Press; 1997. p. 1-41.
2. MacKenzie WR, Schell WL, Blair KA, Addiss DG, Peterson DE, Hoxie NJ, et al. Massive outbreak of waterborne *Cryptosporidium* infection in Milwaukee, Wisconsin: recurrence of illness and risk of secondary transmission. Clin Infect Dis 1995;21:57-62.
3. Navin TR. Cryptosporidiosis in humans: review of recent epidemiologic studies. Eur J Epidemiol 1985;1:77-83.
4. Arrowood MJ. Diagnosis. In: Fayer R, editor. *Cryptosporidium* and cryptosporidiosis. Boca Raton (FL): CRC Press; 1997. p. 43-64.
5. Lindsay DS. Laboratory models of cryptosporidiosis. In: Fayer R, editor. *Cryptosporidium* and cryptosporidiosis. Boca Raton (FL): CRC Press; 1997. p. 209-23.
6. Vetterling JM, Jervis HR, Merrill TG, Sprinz H. *Cryptosporidium wrairi* sp. n. from the guinea pig *Cavia porcellus*, with an emendation of the genus. Journal of Protozoology 1971;18:243-7.
7. Awad-el-Kariem FM, Robinson HA, Dyson DA, Evans D, Wright S, Fox MT, et al. Differentiation between human and animal strains of *Cryptosporidium parvum* using isoenzyme typing. Parasitology 1995;110:129-32.
8. McLauchlin J, Casemore DP, Moran S, Patel S. The epidemiology of cryptosporidiosis: application of experimental sub-typing and antibody detection systems to the investigation of water-borne outbreaks. Folia Parasitol 1998;45:83-92.
9. Nichols GL, McLauchlin J, Samuel D. A technique for typing *Cryptosporidium* isolates. Journal of Protozoology 1991;38:237S-40.
10. Carraway M, Widmer G, Tzipori S. Genetic markers differentiate *C. parvum* isolates. J Eukaryot Microbiol 1994;41:26S.
11. Morgan UM, Constantine CC, O'Donoghue P, Meloni BP, O'Brien PA, Thompson RCA. Molecular characterization of *Cryptosporidium* isolates from humans and other animals using random amplified polymorphic DNA analysis. Am J Trop Med Hyg 1995;52:559-64.

12. Shianna KV, Rytter R, Spanier JG. Randomly amplified polymorphic DNA PCR analysis of bovine *Cryptosporidium parvum* strains isolated from the watershed of the Red River of the North. Appl Environ Microbiol 1998;64:2262-5.
13. Bonnin A, Fourmaux MN, Dubremetz JF, Nelson RG, Gobet P, Harly G, et al. Genotyping human and bovine isolates of *Cryptosporidium parvum* by polymerase chain reaction-restriction fragment length polymorphism analysis of a repetitive DNA sequence. FEMS Microbiol Lett 1996;137:207-11.
14. Widmer G, Tzipori S, Fichtenbaum CJ, Griffiths JK. Genotypic and phenotypic characterization of *Cryptosporidium parvum* isolates from people with AIDS. J Infect Dis 1998;178:834-40.
15. Spano F, Putignani L, McLauchlin J, Casemore DP, Crisanti A. PCR-RFLP analysis of the *Cryptosporidium* oocyst wall protein (COWP) gene discriminates between *C. wrairi* and *C. parvum*, and between *C. parvum* isolates of human and animal origin. FEMS Microbiol Lett 1997;150:209-17.
16. Morgan UM, Constantine CC, Forbes DA, Thompson RCA. Differentiation between human and animal isolates of *Cryptosporidium parvum* using rDNA sequencing and direct PCR analysis. J Parasitol 1997;83:825-30.
17. Peng MM, Xiao L, Freeman AR, Arrowood MJ, Escalante AA, Weltman AC, et al. Genetic polymorphism among *Cryptosporidium parvum* isolates: evidence of two distinct human transmission cycles. Emerg Infect Dis 1997;3:567-73.
18. Chrisp CE, LeGendre M. Similarities and differences between DNA of *Cryptosporidium parvum* and *C. wrairi* detected by the polymerase chain reaction. Folia Parasitol 1994;41:97-100.
19. Carraway M, Tzipori S, Widmer G. Identification of genetic heterogeneity in the *Cryptosporidium parvum* ribosomal repeat. Appl Environ Microbiol 1996;62:712-6.
20. Kilani RT, Wenman WM. Geographical variation in 18S rRNA gene sequence of *Cryptosporidium parvum*. Int J Parasitol 1994;24:303-6.
21. Widmer G. Genetic heterogeneity and PCR detection of *Cryptosporidium parvum*. Adv Parasitol 1998;40:223-39.
22. Cai J, Collins MD, McDonald V, Thompson DE. PCR cloning and nucleotide sequence determination of the 18S rRNA genes and internal transcribed spacer 1 of the protozoan parasites *Cryptosporidium parvum* and *Cryptosporidium muris*. Biochim Biophys Acta 1992;1131:317-20.
23. Le Blancq SM, Khramtsov NV, Zamani F, Upton SJ, Wu TW. Ribosomal RNA gene organization in *Cryptosporidium parvum*. Mol Biochem Parasitol 1997;90:463-78.
24. Johnson DW, Pieniazek NJ, Rose JB. DNA probe hybridization and PCR detection of *Cryptosporidium parvum* compared to immunofluorescence assay. Water Science and Technology 1993;27:77-84.
25. Johnson DW, Pieniazek NJ, Griffin DW, Misener L, Rose JB. Development of a PCR protocol for sensitive detection of *Cryptosporidium* oocysts in water samples. Appl Environ Microbiol 1995;61:3849-55.
26. Tzipori S, Griffiths JK. Natural history and biology of *Cryptosporidium parvum*. Adv Parasitol 1998;40:5-36.
27. Morgan UM, Sargent KD, Deplazes P, Forbes DA, Spano F, Hertzberg H, et al. Molecular characterization of *Cryptosporidium* from various hosts. Parasitology 1998;117:31-7.
28. Sargent KD, Morgan UM, Elliot A, Thompson RCA. Morphological and genetic characterization of *Cryptosporidium* oocysts from domestic cats. Vet Parasitol 1998;77:221-7.
29. Navin TR, Weber R, Vugia DJ, Rimland D, Roberts JM, Addiss DG, et al. Declining CD4+ T-lymphocyte counts are associated with increased risk of enteric parasitosis and chronic diarrhea: results of a 3-year longitudinal study. J Acquir Immune Defic Syndr Hum Retrovirol 1999;20:154-9.
30. Melvin DM, Brooke MM. Laboratory procedures for the diagnosis of intestinal parasites. 3rd ed. Atlanta: Centers for Disease Control; 1982. Publication no. (CDC) 85-8282.
31. Garcia LS, Shum AC, Bruckner DA. Evaluation of a new monoclonal antibody combination reagent for the direct fluorescent detection of *Giardia* cysts and *Cryptosporidium* oocysts in human fecal specimens. J Clin Microbiol 1992;30:3255-7.
32. da Silva AJ, Bornay-Llinares FJ, Moura INS, Slemenda SB, Tuttle JL, Pieniazek NJ. Fast and reliable extraction of protozoan parasite DNA from fecal specimens. Molecular Diagnosis. In press 1999.
33. Schuler GD, Altschul SF, Lipman DJ. A workbench for multiple alignment construction and analysis. Proteins 1991;9:180-90.
34. Felsenstein J. PHYLIP—phylogeny inference package. Cladistics 1989;5:164-6.
35. Roth A, Fischer M, Hamid ME, Michalke S, Ludwig W, Mauch H. Differentiation of phylogenetically related slowly growing mycobacteria based on 16S-23S rRNA gene internal transcribed spacer sequences. J Clin Microbiol 1998;36:139-47.
36. Bornay-Llinares FJ, da Silva AJ, Moura IN, Myjak P, Pietkiewicz H, Kruminis-Lozowska W, et al. Identification of *Cryptosporidium felis* in a cow by morphologic and molecular methods. Appl Environ Microbiol 1999;65:1455-8.

